# Supplementary material for: Production, purification, characterization, and biological properties of Rhodosporidium paludigenum polysaccharide
Source: PLoS One. 2021 Jan 29;16(1):e0246148. doi: 10.1371/journal.pone.0246148 (PMC7845956; doi:10.1371/journal.pone.0246148)
Supplement: Attachment — Submitted filename: 11-23 plos one srpporting information (1).docx [file pone.0246148.s003.docx]

# Supporting information

Characterization and Biological Properties of Marine Red Yeast Rhodosporidium Mucilaginosa Polysaccharide

Mengjian LIU, WenJu ZHANG^*^, Jun YAO, Junli Niu

*Shihezi University, Shihezi, Xinjiang Province, China*

[*zhangwj1022@sina.com*](mailto:zhangwj1022@sina.com)

**Fig 1. Interaction of temperature and pH Fig 2. Interaction of blades speed and pH**

**on the yield of MRYP	on the yield of MRYP**

**Fig 3. Interaction of temperature and**

**blades speed on the yield of MRYP**

Fig. 1, 2, and 3 represented three dimension surface (3D-surface) plot and two dimensional response projection (2D-projection) as a function of temperature, pH and blades speed treatment on matrine yield. The 3D-surface and 2D-projection intuitively showed the response over a region of independent variables and the relationship between experimental levels of each factor. The 3D-plot and 2D-projection of response surface represent the interaction between two factors in matrine production (mg/L) by keeping the other two variables constant: temperature (℃) and pH (A), blades speed (r/min) and pH (B), blades speed (r/min) and temperature (℃) (C), (the yellow and red dot represent design points below predicted value and above predicted value, respectively).

Fig. 1 showed the effect of temperature and pH on MRYP yield. The 3D-surface showed evidence that marine yield increased upon increasing temperature and pH. A maximal MRYP production was obtained when the temperature was 30.18 ℃ and pH was 6.72. Afterwards, the MRYP production decreased with temperature and pH increase. Fig. 2 showed that increasing the blades speed to more than 461.36 r/min caused shrink of MRYP yield. A quadratic effect of blades speed and temperature in the response was observed in Fig. 3. The curves of Fig. 3 bear some similarity to that in Fig. 1 and 2, respectively.

Thus, the best content of temperature, pH, and blades speeds were found to be 30.18 ℃, 6.72, and 461.36 r/min, respectively. The predicted MRYP yield obtained from the model using the optimal medium was 4876.80 mg/L.

**Fig 4. The MRYP yields of optimized and pre-optimization**

Time-course process of batch fermentation in a 12 L bioreactor for MRYP production of optimized and pre-optimization. After 72h fermentation, the average result of verification tests was 4323.90 mg/L, which was 1.31 times higher than that the yield before optimization, which was 3300.00 mg/L.

**Fig 5. Linear elution figure of pure polysaccharides**

To obtain high-purity polysaccharide products for structural elucidation, the MRYP was fractionated by Cellulose DEAE-52 column. The elution profile indicated that MRYP was isolated to two independent elution peaks of MYH-1 and MYH-2 with yields of 53.60% and 28.75%, respectively. MYH-1 and MYH-2 did not contain fat and protein contents were below the detection limit.

**Fig 6. HPGPC chromatograms of MYH-1 and MYH-2**

MYH-1 and MYH-2 showed only one single and symmetrical narrow peak with elution time of about 14.432 min and 20.587 min in the HPGPC chromatogram, respectively. This result indicated that MYH-1 and MYH-2 were homogeneous polysaccharide, without fat and protein contents were below the detection limit. Based on the calibration with standard dextrans, the average molecular weights of MYH-1 and MUH-2 were estimated to be about 246.92 kDa and 21.88 kDa, respectively.

**Fig 7. Standard curve of polysaccharide of the standard molecular weight**

This standard curve was a log-log plot, which was made by standard dextrans (404 kDa, 212k Da, 112 kDa, 47.3 kDa, 22.8 kDa, 11.8 kDa, 5.9 kDa, and 2.5 kDa). The regression equation as follows: Y=7E+09e^-0.67x^, with R^2^=0.9943. The X means retention time, and the Y means molecular weight of polysaccharide.

**Fig 8. Standard curve of glucose and mannose**

The standard curves of glucose and mannose were made by concentration of the standard solution of pure mannose and glucose (200 μg/mL, 400 μg/mL, 600 μg/mL, 800 μg/mL, and 1000 μg/mL). The regression equations of mannose and glucose as follow: Y=0.0194X+0.6695 with R^2^=0.9954, and Y=0.0159X+0.3315 with R^2^=0.9978. The concentrations of mannose and glucose in MYH-1 or MYH-2 were be detected by the peak areas in HPLC.

**Fig 9. HPLC chromatogram of glucose**

**Fig 10. HPLC chromatogram of mannose**

The HPLC profiles of monosaccharide standards were made by pure mannose and glucose solutions (200 μg/mL), separately. The operation conditions of HPLC were as follows: mobile phase was deionized water; flow velocity was 0.5mL/min; column temperature was 85 ℃; and the sample size was 50 μL. The first peaks were PMP in Fig 9 and Fig 10, and the retention time were 10.294 min and 10.317 min, separately. The second peaks were glucose and mannose in Fig 9 and Fig 10, and the retention time were 26.384 min and 30.217 min, separately.

**Fig 11. HPLC chromatogram of the MYH-1 hydrolysate**

There were three main peaks in this HPLC chromatogram of MYH-1. The peaks were PMP, glucose and mannose. The retention time of PMP, glucose and mannose were 10.328 min, 26.381 min and 30.107 min, which nearly same as HPLC chromatogram of monosaccharide standards. The concentration and proportion of glucose and mannose in MYH-1 were 938.72 μg/ml,769.15 μg/ml, and 46.94%, 38.46%, respectively. The operation conditions of HPLC were same as the conditions of HPLC chromatogram of monosaccharide standards.

**Fig 12. HPLC chromatogram of the MYH-2 hydrolysate**

As the same as Fig 11, there were three main peaks in this HPLC chromatogram of MYH-2. The retention time of PMP, glucose and mannose were 10.360 min, 26.270 min and 30.096 min. The concentration and proportion of glucose and mannose in MYH-2 were 1341.97 μg/mL, 143.46 μg/mL, and 67.10%, 7.17%, respectively. The operation conditions of HPLC were same as the conditions of HPLC chromatogram of monosaccharide standards.

**Fig 13. Scavenging capacities for** ·**DPPH,** ·**OH, and** ·**O_2_^–^ of MRYP**

The scavenging effects of MRYP are shown in Fig 13. Three curves represented the clearance rate of MRYP on ·DPPH, ·OH, and ·O2–, and MRYP scavenged radicals for three free radicals in a dose-dependent manner.  The MRYP scavenging of ·DPPH, ·OH, and ·O_2_– reached a peak, which were 32.26%, 24.34%, and 22.09%, respectively, while the concentration of MRYP was 1000 μg/mL.
