# Supplementary material for: Production, purification, characterization, and biological properties of Rhodosporidium paludigenum polysaccharide
Source: PLoS One. 2021 Jan 29;16(1):e0246148. doi: 10.1371/journal.pone.0246148 (PMC7845956; doi:10.1371/journal.pone.0246148)
Supplement: Attachment — Submitted filename: Response to Reviewers 1-7.doc [file pone.0246148.s004.doc]

Suggestions on revision and changes for reviewers

| Location | The original | Expert opinion | Modification or explanation |
| --- | --- | --- | --- |
| In all article |  | The language quality has still plenty of scope for improvement. | The whole article has been improved by native English professer. |
| Line 25 | Marine red rhodosporidium paludigenum polysaccharide | long keyword 'Marine red yeast rhodosporidium paludigenum polysaccharide' | Has been changed. ‘Rhodosporidium paludigenum polysaccharide’ |
| Line 292-294 |  | Table 2. 'polysaccharide' should be added to the title and a,b,c should be explained on the legend | Values are means ± standard deviations. In the same row, values with different small letter superscripts mean significant difference (P<0.05), different capital letter superscripts mean significant difference (P<0.01). The same as below. The same as below. |
| Line505 | Over more | English errors such as 'Over more' in line 518 | Furthermore |
